# *Taenia solium* cysticercosis and taeniosis: Achievements from the past 10 years and the way forward

**DOI:** 10.1371/journal.pntd.0005478

**Published:** 2017-04-20

**Authors:** Hélène Carabin, Andrea S. Winkler, Pierre Dorny

**Affiliations:** 1 Department of Biostatistics and Epidemiology, College of Public Health, University of Oklahoma Health Sciences Center, Oklahoma City, Oklahoma, United States of America; 2 Centre for Global Health, Institute of Health and Society, Faculty of Medicine, University of Oslo, Oslo, Norway; 3 Department of Neurology, Technical University of Munich, Munich, Germany; 4 Unit of Veterinary Helminthology, Department of Biomedical Sciences, Institute of Tropical Medicine Antwerp, Antwerp, Belgium; Baylor College of Medicine, Texas Children's Hospital, UNITED STATES

**Fig 1:**
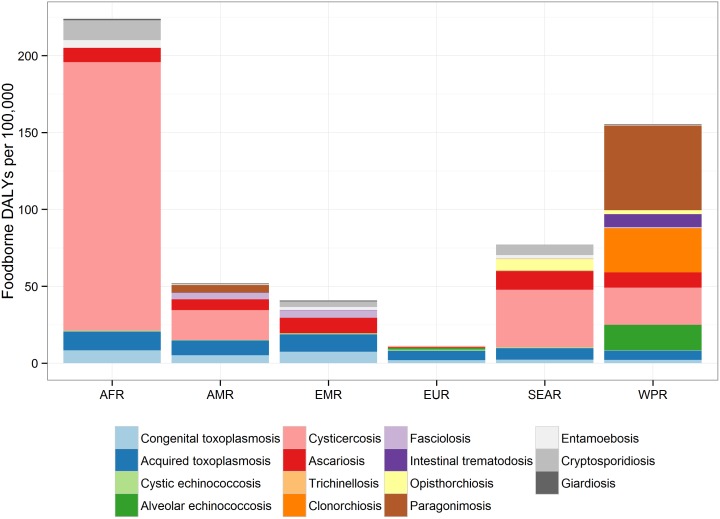
Contribution of each parasite to foodborne disability adjusted life years in regions: The relative contribution to the DALY incidence by each agent for each of the regions. This includes enteric protozoa to complete the picture on foodborne parasitic diseases. The figure, title and legend were taken from Figure 4 in [2] (http://dx.doi.org/10.1371/journal.pmed.1001920.g002). No changes were made to the original figure, title and legend in [2].

*Taenia solium* is a cestode with humans acting as the definitive hosts and pigs as the intermediate hosts. The infection is endemic in areas where sanitation is poor, pigs scavenge for food and veterinary meat inspection is limited. In addition to the economic losses farmers face from selling infected pork, cysticercosis incurs a significant public health burden when humans ingest parasite eggs, which liberate larvae that may establish in the nervous system, a condition called neurocysticercosis (NCC). The past 10 years have seen two independent groups publish, for the first time, NCC-associated epilepsy Disability Adjusted Life Years (DALYs) estimates. The 2010 NCC-associated DALYs was estimated at 0.5 million (Uncertainty Interval (UI: 0.38 to 0.66 million) by the Global Burden of Disease [1], while the World Health Organization’s Foodborne Disease Burden Epidemiology Reference Group obtained a much larger estimate at 2.8 million (UI: 2.1 to 3.6 million), making NCC-associated epilepsy the parasitic foodborne infection with the largest number of DALYs globally (Fig 1) [2]. While such estimates are a step in the right direction, these contrasting results and the exclusion of NCC-associated sequelae other than epilepsy underscore great uncertainties remaining around the epidemiology and pathology of NCC.

**Fig 2:**
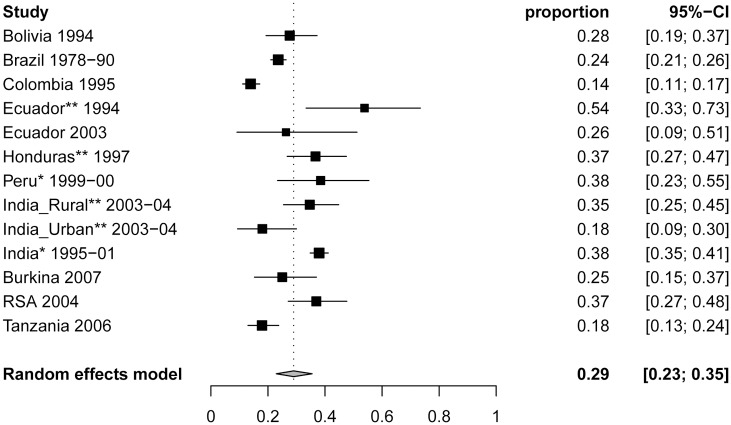
Forest plots of the proportion of NCC (95% CI) in people with epilepsy from 12 studies reporting from cases in all age groups. *Indicates studies among people with epilepsy and seizures. ** Indicates studies among people with active epilepsy only. The figure, title and legend were taken from Figure 4 in [5] (http://dx.doi.org/10.1371/journal.pntd.0000870.g004). No changes were made to the original figure, title and legend in [5].

The availability of an increasing number of clinical and epidemiological studies on NCC and cysticercosis has resulted in the publication of several systematic reviews and meta-analyses in the past 10 years [3–6]. These systematic reviews have confirmed long-held beliefs that approximately 30% of people living with epilepsy in countries endemic for cysticercosis show NCC lesions in their brain (Fig 2)[5]. There is also evidence of increasing NCC cases being diagnosed in non-endemic areas such as Europe and the Unites States. NCC is now increasingly recognized as a problem not confined to rural areas but also found in urban centers[7]. Supportive evidence for perilesional edema around calcified lesions and hippocampal sclerosis has improved our understanding of NCC pathology and has had new implications for treatment. Up to recently, NCC treatment approach was very much left to the attending physician [3]. However, new evidence suggests that anti-parasitic and anti-inflammatory medication in active symptomatic NCC are beneficial to control seizures during treatment and thereafter [8, 9]. Combined treatment with albendazole and praziquantel was newly found to lead to more efficacious cyst clearance and better seizures prognosis compared to albendazole alone [10]. While NCC management guidelines for parenchymal NCC were published in 2013 [11], new updated guidelines for resource-rich and resource-poor countries are underway. In addition to epilepsy, NCC can manifest itself with a plethora of neurological signs and symptoms, including headaches and psychiatric disorders [3, 4]. Recent debates have also focused on management of the clinically more severe extraparenchymal NCC for which HP10 antigen in cerebrospinal fluid has been suggested as a marker [12]. Spinal NCC also seems more frequent than previously assumed, especially among subarachnoid NCC cases [3]. The impact that HIV infection and initiation of anti-retroviral therapy have on the clinical presentation of NCC, its immunodiagnosis and treatment are other areas which need further exploration. Unfortunately, the occurrence of clinical signs and symptoms and of asymptomatic NCC remains poorly described due to the diagnostic challenges of brain infections, an issue which was identified as one of the greatest challenges to advances in infectious disease of the brain research [13]. Indeed, NCC diagnosis relies on brain imaging which is often unavailable to populations living in endemic areas. However, biomarkers for parenchymal NCC are being discussed and developed [14, 15] while new fusion techniques for combining CT and MRI images are being suggested for better visualization of NCC lesions [16].

Several researchers have been developing alternatives to the still preferred, yet complex and expensive, enzyme linked immunoelectrotransfer blot (EITB) assay for the immunodiagnosis of cysticercosis [17]. The most promising genes encoding diagnostic antigens are members of the 8 kDa family, used as fusion proteins/peptides, and recombinant proteins derived from the glycoprotein T24 and GP50, used independently or as antigen cocktail. *T*. *solium*- specific copro-antigen ELISA, Real-time polymerase chain reaction (RT-PCR) on stool extracts and serological diagnosis using the recombinant rES33 antigen have also been developed. A point of care test for simultaneous taeniosis and cysticercosis diagnosis with rES33 and rT24 antigens is currently being field evaluated. In contrast to developments of human *T*. *solium* infections diagnostic tools, diagnosis of porcine cysticercosis has not progressed much although accurate diagnosis is needed for food safety, epidemiological studies and public health intervention monitoring. While current antibody detection methods overestimate the prevalence due to transient antibodies, antigen detection methods show poor specificity due to cross reactions with *T*. *hydatigena*. Currently, laborious and expensive full carcass detection remains the only reliable method calling for urgent development of pen-side tests [18].

There has been an increasing understanding of the immunological response to NCC in the past 10 years. Viable cysticerci has been shown to provoke a downregulation of inflammatory Th1 response and a switch to Th2 cytokines associated with asymptomatic disease, while a Th1 host response predominantly follows the degeneration of the cysts [15]. Cytokines, other immune mediators and enzymes are important in driving the pro-inflammatory response and increased permeability of the blood brain barrier. In addition, the conduct of several cohort studies of NCC cases and of individuals living in endemic communities have improved our knowledge of the epidemiology and pathogenesis of cysticercosis and of the interpretation of immunodiagnostic test results. In endemic regions, many people have transient antibodies resulting from exposure to oncospheres without cysticerci establishment, while the significance of transient antigens has yet to be demonstrated [17]. The result is that serological tests overestimate the true prevalence of cysticercosis.

A recent systematic review of the frequency of and risk factors associated with human cysticercosis and taeniosis has emphasized the clustered nature of this infection and very large between- and within- country variations in sero-prevalence estimates depending on which communities are selected for study. Human active cysticercosis prevalence has been reported to vary from 0.6% in Ha Tinh, Viet Nam to 21.6% in one Democratic Republic of Congo community [6]. The reported prevalence of *T*. *solium* taeniosis is generally lower, but this could be due to the two-step approach needed to confirm microscopy- or copro-antigen-positive samples with RT-PCR. The new rES33 test is likely to improve our understanding of the prevalence and factors associated with *T*. *solium* taeniosis. Several studies have identified older men as more often infected with cysticerci [6]. The effect of other risk factors such as pig management practices and factors affecting the survival of eggs in the environment remain to be confirmed. The development of more affordable tools to detect eggs in the environment is likely to improve our knowledge of the relative contribution of taeniosis auto-infection, contact with a taeniosis carrier and infection through the ingestion of eggs in the environment have on cysticercosis and NCC.

The past 10 years have also seen several research initiatives to control the infection through community-based interventions [19, 20]. Unfortunately, control groups, randomization, or sample sizes with sufficient power to detect significant differences for intervention implemented at the community level have rarely been used, leaving an important gap in knowledge as to what intervention may be effective at controlling the infection on a large scale. A recently published large scale community-based trial showed that a combination of human and pig mass drug administration treatment may not be enough to eliminate the infection [21], suggesting that a One Health approach, including treatment and/or vaccination and better management of pigs, treatment and education of humans and the building and use of latrines, is needed to control and potentially eliminate cysticercosis. Recently funded One Health initiatives across the globe demonstrate a strong desire to follow the 2030 Sustainable Development Goals of ending epidemics of neglected tropical diseases such as cysticercosis.
